# Supplementary material for: Garnet microstructures suggest ultra-fast decompression of ultrahigh-pressure rocks
Source: Nat Commun. 2023 Sep 27;14:6012. doi: 10.1038/s41467-023-41310-w (PMC10533891; doi:10.1038/s41467-023-41310-w)
Supplement: Supplementary file 1 — Supplementary Information [file 41467_2023_41310_MOESM1_ESM.pdf]

Garnet microstructures suggest ultra-fast decompression of ultrahigh-pressure rocks

Luisier Cindy [1], Tajčmanová Lucie [2], Yamato Philippe [3,1], Duretz Thibault [1]

1 Institute of Geosciences, Goethe-University Frankfurt, Frankfurt am Main, Germany

2 Institute of Earth Sciences, Heidelberg University, Heidelberg, Germany

3 Univ Rennes, CNRS, Géosciences Rennes, Rennes, France

Supplementary information

All the figures presented in the supplementary material as well as Fig. 4 in the main text were made with Makie[1].

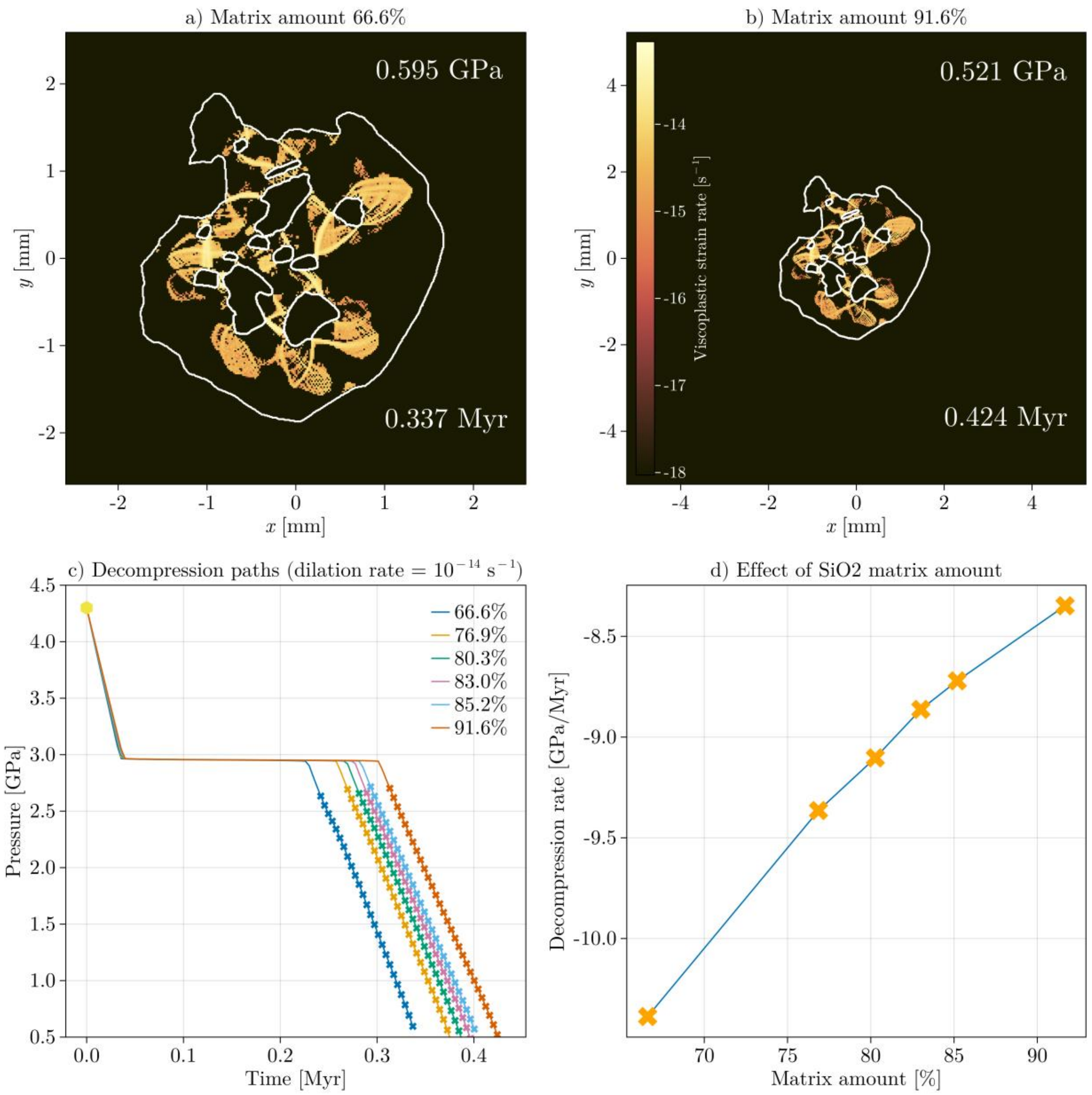

Figure S1: Effect of matrix amount

a) and **b)** Simulations were run for a variable percentage of SiO_2 matrix (66.6% to 91.6%). **c)** The results show that a larger amount of matrix allows to extend the duration of the decompression, thus decreasing the magnitude of the apparent decompression rate. Varying the matrix amount did not impact pressure at which yielding initiated (~2.7 GPa, see c). For the same pressure, the shear banding was also identical (a and b). **d)** The simulation with 91.6% matrix exhibited an apparent decompression rate of -8.5 GPa/Myr, while the model with 66.6% matrix leads to -12 GPa/Myr.

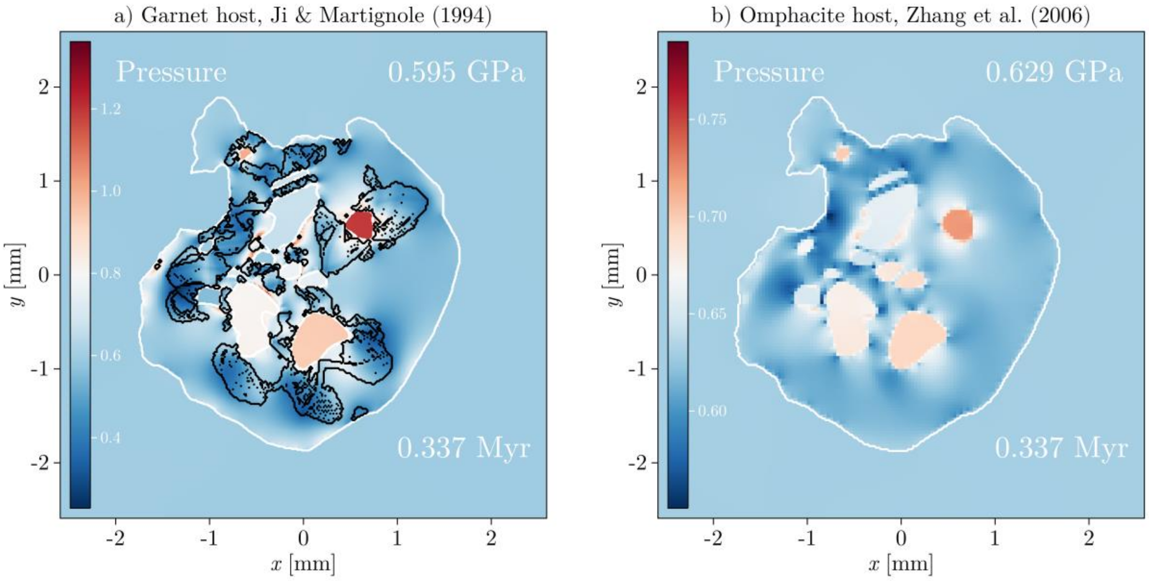

Figure S2: Effect of host rheology

Comparison of simulation results using different host creep laws: **a)** garnet[2] and **b)** omphacite[3]. The colormap correspond to the pressure field, the white contour denotes the material boundaries and the black contour indicates the frictional plastic zone. An omphacite creep law does not allow to build sufficient deviatoric stress and does not trigger frictional plasticity of the host, despite the phase transitions that occurred in both the matrix and inclusion.

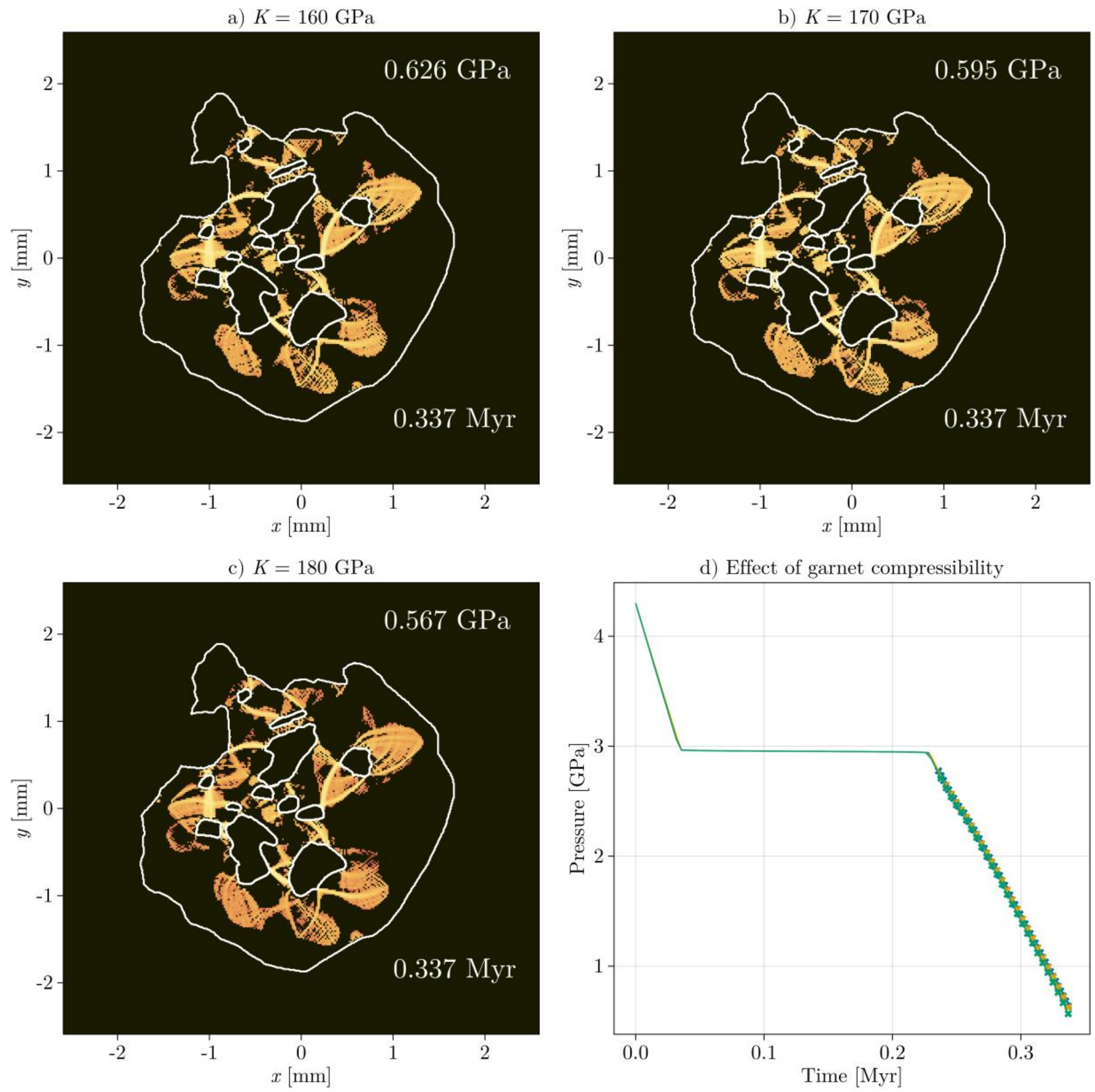

Figure S3: Effect of garnet compressibility
The garnet compressibility (K) was varied between **a)** 160 GPa, **b)** 170 GPa and **c)** 180 GPa. **d)** This parameter variation had a very limited impact on our result. The timing, pressure and patterns of shear banding were impacted minimally.

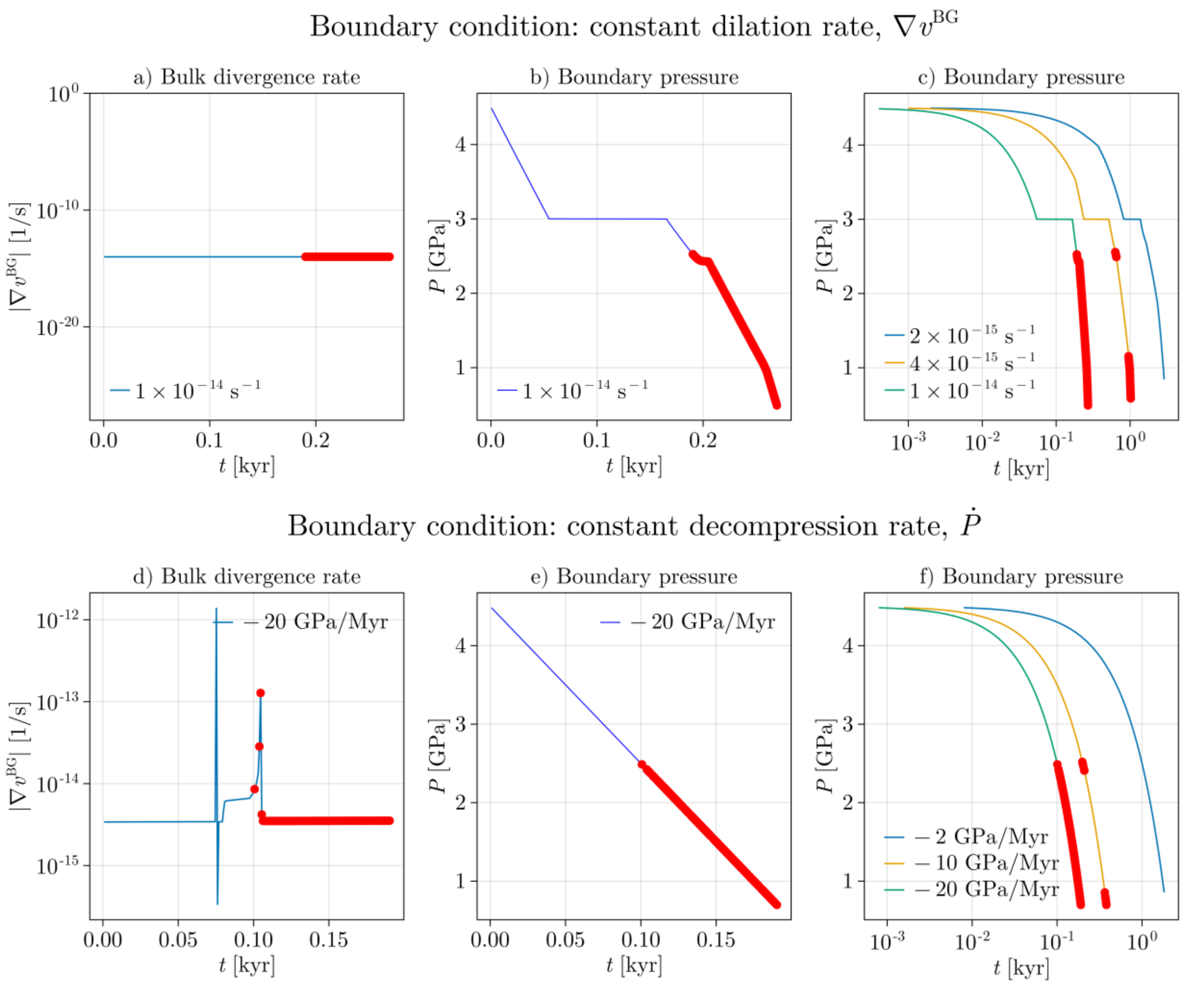

Figure S4: Effect of boundary conditions.

To study the effect of boundary condition, we designed simplified 1D radial models of visco-elasto-plastic host-inclusion-matrix decompression. **a), b), c)** The results show that, with a constant boundary dilation rate, buffering of boundary pressure takes place during phase transformation. **d), e), f)** On the contrary, if a boundary decompression rate is applied, the boundary divergence can vary over several orders of magnitude to accommodate the phase transition. Both types of boundaries predict that fast apparent decompression rates are needed to explain frictional yielding of garnet. The constant dilation rate condition predicts slightly lower apparent decompression rate due to the transient pressure buffering effect that delays matrix decompression. For constant dilation rates and a matrix amount of 66.6%, frictional yielding was attained for apparent decompression rates of ~10 GPa/Myr. Models with constant decompression required decompression rates larger than 15 GPa/Myr. The variations in shear banding patterns could not be investigated due to the 1D nature of the models. On all panels, the red dots indicate conditions for which frictional plastic yielding is taking place.

Supplementary Table 1 Parameters used in the 2D thermo-mechanical model, parameters with * are after ref.[4].

Parameter	Symbol	Units	Whole model	
Temperature	T	K	$T = (1/(1.02\times10^{-2})) * \ln(P/(2.523\times10^{6})) + 273.15$	
Cohesion	C	Pa	10^{7}	
Visco-plastic modulus	η^{VP}	Pa·s	5×10^{19}	
			**SiO_2**	**Garnet**
Pre-exponential factor	A	$Pa^{-n}\cdot s^{-1}$	2.667×10^{-20}	2.7952×10^{-7}
Exponent	n	-	2.4	2.22
Activation energy	Q	$J\cdot mol^{-1}$	156.0×10^{3}	485.0×10^{3}
Activation volume	V	$m^{3}\cdot mol^{-1}$	0	10^{-5} *
Density model	ρ	$kg\cdot m^{-3}$	$\rho = f(P,T)$	$\rho = \rho_0 \exp(P/K-\alpha T)$
Bulk modulus	K	Pa	Holland and Powell, 2011	1.7×10^{11}
Thermal expansivity	α	K^{-1}	Holland and Powell, 2011	3×10^{-5}
Reference density	ρ_0	$kg\cdot m^{-3}$	Holland and Powell, 2011	3577
Friction angle	φ	°	35	20
Dilation angle	Ψ	°	10	5
Shear modulus	G	Pa	4×10^{10}	9×10^{10}

Supplementary References

1. Danisch, S. & Krumbiegel, J. Makie.jl: Flexible high-performance data visualization for Julia. *Journal of Open Source Software* **6**(65), 3349 (2021) doi:10.21105/joss.03349
2. Ji, S. & Martignole, J. Ductility of garnet as an indicator of extremely high temperature deformation. *Journal of Structural Geology* **16**, 985–996 (1994).
3. Zhang, J. Green II, H. W. & Bozhilov, K. N. Rheology of omphacite at high temperature and pressure and significance of its lattice preferred orientations. *Earth and Planetary Science Letters* **246**(3-3), 432-443 (2006).
4. Mei, S. Suzuki, A. M., Kohlstedt, D. L. & Xu, L. Experimental investigation of the creep behavior of garnet at high temperatures and pressures. *Journal of Earth Science* **21**, 532-540 (2010).
